# Impaction of suction catheter – Complication of endotracheal suctioning

**DOI:** 10.4103/0972-5229.76092

**Published:** 2010

**Authors:** Aikta Gupta, Anup Mohta, Geeta Kamal, Sapna Bathla

**Affiliations:** **From:** Department of anesthesia, CNBC Geeta colony, Delhi, India; 1 Department of paediatric surgery, CNBC Geeta colony, Delhi, India

Dear Editor,

Endotracheal tube (ETT) suction is necessary to clear secretions and to maintain airway patency, and to optimize oxygenation and ventilation in an intubated patient. The goal of ETT suction should be to maximize the amount of secretions removed with minimal adverse effects. We report a case of suction catheter impaction during endotracheal suction.

A 2-year-old male child was scheduled for exploratory laprotomy for perforation peritonitis. Preoperatively, the patient was ASA grade 1 with laboratory investigations within normal limits. With all the preoperative necessary arrangements, the patient was taken up for emergency surgery. The patient was induced and intubated with uncuffed 4.5 ETT. The surgery was uneventful and it lasted for 1 hour. At the end of the operation, reversal was given after confirming the spontaneous efforts. Just before extubation, crackling sounds were heard from the ETT, which were confirmed on auscultation. Suction catheter Fr G no.8 was introduced and as we were withdrawing the catheter, resistance was encountered resulting in inability to either advance or withdraw the catheter. Gentle traction on the catheter failed to retrieve the impaction. As all our efforts failed and the patient saturation started falling, we decided to extubate the child with suction catheter *in situ*. After extubation, we gently did mask ventilation with 100% oxygen and within seconds saturation picked up. After extubation, on repeat attempt to remove the suction catheter from ETT, slight resistance was encountered but we were successful in removing it. On visual inspection, viscous secretions were observed on the outer aspect of suction catheter, which were getting adhered to the inner surface of the tube and probably thereby preventing its removal.

We would like to bring to the attention of our readers that this problem can routinely be faced by anesthesiologists and intensivists. In difficult airway cases, this can be life threatening.

There have been previous reports of nasogastric tube causing extraluminal ETT obstruction.[1,2] The author reported incidental suction catheter impaction followed by its division into two pieces on pulling vigorously in preformed nasotracheal tube in a pediatric dental patient.[3] Jagannathan *et al*. reported endotracheal obstruction by suction catheter passing into the murphy eye of the tube.[4]

To our knowledge, this is the first reported case of impaction of suction catheter within ETT even though the catheter was of recommended size and there was no knotting inside the ETT (unlike Jagannathan *et al*.)

In this regard, we presume that lubrication of suction catheter can be made a routine practice, especially in children as most of the time they are premedicated with injection glycopyrolate which makes secretions thick and viscous. But more research is needed to support routine practice of lubrication of suction catheter in children.
